# Emergency department triage prediction of clinical outcomes using machine learning models

**DOI:** 10.1186/s13054-019-2351-7

**Published:** 2019-02-22

**Authors:** Yoshihiko Raita, Tadahiro Goto, Mohammad Kamal Faridi, David F. M. Brown, Carlos A. Camargo, Kohei Hasegawa

**Affiliations:** 1 Department of Emergency Medicine, Massachusetts General Hospital, Harvard Medical School, 125 Nashua Street, Suite 920, Boston, MA USA; 2 Graduate School of Medical Sciences, The University of Fukui, Fukui, Japan

**Keywords:** Triage, Emergency department, Prediction, Machine learning, Mortality, Critical care, Hospitalization, Hospital transfer, Decision curve analysis

## Abstract

**Background:**

Development of emergency department (ED) triage systems that accurately differentiate and prioritize critically ill from stable patients remains challenging. We used machine learning models to predict clinical outcomes, and then compared their performance with that of a conventional approach—the Emergency Severity Index (ESI).

**Methods:**

Using National Hospital and Ambulatory Medical Care Survey (NHAMCS) ED data, from 2007 through 2015, we identified all adult patients (aged ≥ 18 years). In the randomly sampled training set (70%), using *routinely* available triage data as predictors (e.g., demographics, triage vital signs, chief complaints, comorbidities), we developed four machine learning models: Lasso regression, random forest, gradient boosted decision tree, and deep neural network. As the reference model, we constructed a logistic regression model using the five-level ESI data. The clinical outcomes were critical care (admission to intensive care unit or in-hospital death) and hospitalization (direct hospital admission or transfer). In the test set (the remaining 30%), we measured the predictive performance, including area under the receiver-operating-characteristics curve (AUC) and net benefit (decision curves) for each model.

**Results:**

Of 135,470 eligible ED visits, 2.1% had critical care outcome and 16.2% had hospitalization outcome. In the critical care outcome prediction, all four machine learning models outperformed the reference model (e.g., AUC, 0.86 [95%CI 0.85–0.87] in the deep neural network vs 0.74 [95%CI 0.72–0.75] in the reference model), with less under-triaged patients in ESI triage levels 3 to 5 (urgent to non-urgent). Likewise, in the hospitalization outcome prediction, all machine learning models outperformed the reference model (e.g., AUC, 0.82 [95%CI 0.82–0.83] in the deep neural network vs 0.69 [95%CI 0.68–0.69] in the reference model) with less over-triages in ESI triage levels 1 to 3 (immediate to urgent). In the decision curve analysis, all machine learning models consistently achieved a greater net benefit—a larger number of appropriate triages considering a trade-off with over-triages—across the range of clinical thresholds.

**Conclusions:**

Compared to the conventional approach, the machine learning models demonstrated a superior performance to predict critical care and hospitalization outcomes. The application of modern machine learning models may enhance clinicians’ triage decision making, thereby achieving better clinical care and optimal resource utilization.

**Electronic supplementary material:**

The online version of this article (10.1186/s13054-019-2351-7) contains supplementary material, which is available to authorized users.

## Background

Over the past two decades, the number of emergency department (ED) visits has increased by approximately 50% in the USA, with 138 million visits in 2014 [1]. This increase has contributed to ED crowding and delays in care [2–4]. The literature has demonstrated that delay in care results in greater morbidity and mortality for many disease conditions [3–7]. ED triage presents the first opportunity to promptly identify high-risk patients and efficiently allocate finite ED resources. Among various triage algorithms, the Emergent Severity Index (ESI) is the most commonly used algorithm in US EDs [8–10]. Despite its wide adoption, it heavily relies on clinical judgment, leading to high inter-rater variability and suboptimal predictive ability [9–13].

The advent of machine learning models has shown promise to improve predictive ability in various conditions (e.g., sepsis, unplanned transfers to intensive care unit) [14–16]. These approaches offer advantages in that they account for high-order, non-linear interactions between predictors and gain more stable prediction [17]. Recent studies have reported that the application of machine learning models may provide high predictive ability at ED triage in selected patient populations and settings—e.g., children [18], patients with asthma and COPD exacerbation [19], and in few urban EDs [11, 20, 21]. Despite this clinical and research promise, no study has yet examined the utility of modern machine learning models for predicting clinical outcomes in a large population of adult patients in the ED.

To address this knowledge gap, we used large ED visit data to develop machine learning models—by using *routinely* available triage data—to accurately predict clinical outcomes after ED triage. We also examined the predictive performance of these models in comparison to the model using the conventional five-level ESI algorithm.

## Methods

### Study design and setting

We used combined data from the ED component of the 2007–2015 National Hospital and Ambulatory Medical Care Survey (NHAMCS) [22]. NHAMCS collects a nationally representative sample of visits to non-institutional general and short-stay hospitals, excluding federal, military, and Veterans Administration hospitals, in the 50 states and the District of Columbia. The survey has been conducted annually since 1992 by the National Center for Health Statistics (NCHS). For example, a total of 21,061 ED visits were surveyed in 2015 and submitted electronically from 267 EDs, equivalent to a weighted national sample of 137 million ED visits. The details of the NHAMCS methods and procedures may be found in the NHAMCS data file [22]. We followed the reporting guideline from the TRIPOD (Transparent Reporting of a multivariable prediction model for Individual Prognosis Or Diagnosis) statement [23]. The institutional review board of Massachusetts General Hospital waived the review of this study.

### Study samples

We identified all adult ED visits (aged ≥ 18 years) recorded in the 2007–2015 data. The study period was chosen because the information on respiratory rates and oxygen saturation levels were not available before 2007. We excluded patients with death on ED arrival, those who left before being seen or against medical advice, and those with missing information or data inconsistencies (i.e., systolic blood pressure > 300 mmHg, diastolic blood pressure > 200 mmHg, pulse rate > 300/min, respiratory rate > 80/min, oxygen saturation > 100%).

### Predictors

As the predictors for the machine learning models, we included *routinely* available information at ED triage settings—i.e., patient age, sex, mode of arrival (walk-in vs. ambulance), triage vital signs (temperature, pulse rate, systolic and diastolic blood pressure, respiratory rate, and oxygen saturation), chief complaints, and patient comorbidities. Chief complaints were reclassified according to the *Reason for Visit Classification for Ambulatory Care* provided [22]. As the comorbidity classification, we adopted 30 Elixhauser comorbidity measures using the data of *the International Classification of Diseases, Ninth Version, Clinical Modification* (*ICD-9-CM*) codes [24, 25].

### Outcomes

The primary outcome was critical care outcome, defined as either direct admission to an intensive care unit (ICU) or in-hospital death, as done in previous studies [11, 12, 19, 26]. The prompt and accurate prediction of the critical care outcome at ED triage enables clinicians not only to efficiently allocate ED resources but also to urgently intervene on high-risk patients. The secondary outcome was hospitalization, defined as either an admission to an inpatient care site or direct transfer to an acute care hospital [11, 12, 19].

### Statistical analysis

In the training set (70% randomly selected samples), we developed a reference model and four machine learning models for each outcome. As the reference model, we fitted a logistic regression model using the conventional ESI as the predictor. NHAMCS uses the five-level ESI algorithm: immediate (level 1), emergent (level 2), urgent (level 3), semi-urgent (level 4), and non-urgent (level 5) [8]. While 7% of the EDs participating in the NHAMCS did not use this classification, NCHS systematically recoded all data into these five levels [22]. We also fitted logistic regression models using demographic and physiologic variables in the NHAMCS data (i.e., age, mean blood pressure, heart rate, and respiratory rate) and APACHE II scoring system [27] as physiologic score-based models.

Next, using machine learning approaches, we developed four additional models: (1) logistic regression with Lasso regularization (Lasso regression), (2) random forest, (3) gradient boosted decision tree, and (4) deep neural network. First, Lasso regularization is one of the models that shrinks regression coefficients toward zero, thereby effectively selecting important predictors and improving the interpretability of the model. Coefficients of Lasso regression are the values that minimize the residual sum of square plus shrinkage penalty [17, 28, 29]. We used a 10-fold cross-validation to yield the optimal of regularization parameter (lambda) minimizing the sum of least square plus shrinkage penalty by using R *glmnet* package [28, 30]. Second, random forest is an ensemble of decision trees from bootstrapped training samples, and random samples of a certain number of predictors are selected to tree induction. We used R *ranger* and *caret* packages to construct random forest models [31, 32]. Third, gradient boosted decision tree is also an ensemble method which constructs new tree models predicting the errors and residuals of previous models. When adding the new models, this model uses a gradient descent algorithm to minimize a loss function [33]. We used R x*gboost* package to construct gradient boosted decision tree models [34]. Lastly, deep neural network model is composed of multiple processing layers. Outcomes are modeled by intermediate hidden units, and each hidden unit consists of the linear combination of predictors which are transformed into non-linear functions [17]. We used six-layer feedforward model with adaptive moment estimation optimizer and tuned hyperparameters (e.g., the number of hidden units, batch size, learning rate, learning rate decay, and dropout rate) using R *Keras* package [35, 36]. In these machine learning models, we used several methods to minimize potential overfitting—e.g., (1) Lasso regularization, (2) out-of-bag estimation, (3) cross-validation, and (4) dropout, Ridge regularization, and batch normalization in each model. To examine the importance of each predictor in the random forest models, we used permutation-based variable importance that is determined by the normalized average value of difference between prediction accuracy of the out-of-bag estimation and that of the same measure after permutating each predictor. In the gradient boosting decision tree models, we also computed the importance that is summed over iterations [32].

In the test set (the remaining 30% sample), we computed the prediction performance of each model that was derived above. As the prediction performance, we computed (1) the area under the receiver-operating-characteristics curve (AUC), (2) net reclassification improvement, (3) confusion matrix results (i.e., sensitivity, specificity, positive predictive value, and negative predictive value), and (4) net benefit through decision curve analysis. To compare the receiver-operating-characteristics curve (ROC) between models, Delong’s test was used [37]. The net reclassification improvement was used to quantify whether a new model provides clinically relevant improvements in prediction [38]. The decision curve analysis incorporates the information about the benefit of correctly triaging patients (true positives) and the relative harm of the over-triages (false positives)—i.e., the net benefit—over a range of threshold probability of the outcome (or clinical preference) [39–42]. We graphically demonstrated the net benefit of each model through a range of threshold probabilities of the outcome as a decision curve. All analyses were performed with R version 3.5.1.

## Results

During 2007–2015, the NHAMCS recorded 209,800 adult ED visits. Of these, we excluded 97 ED visits with death on arrival, 6350 visits who left before being seen, or against medical advice, 67,674 visits with missing information, and 209 visits with data inconsistencies, leaving the analytic cohort of 135,470 ED visits. The patient characteristics between the analytic and non-analytic cohorts were generally similar (Additional file 1). In the analytic cohort, the median age was 46 years (IQR 29–60 years) and 43.1% were women (Table 1). Overall, 2782 ED visits (2.1%) had critical care outcome; pneumonia, chest pain, acute cerebrovascular disease, and congestive heart failure are the most common diagnoses (Table 2). Additionally, 22,010 ED visits (16.2%) had hospitalization outcome; nonspecific chest pain, abdominal pain, pneumonia, and other lower respiratory diseases are the most common diagnoses.

**Table 1:** Predictor variables and outcomes in 135,470 adult emergency department visits

Variable	*n* = 135,470	
Age (year), median (IQR)	46	(29–60)
Female sex	58,450	(43.1)
Mode of arrival		
Ambulance	26,820	(19.8)
Emergency Severity Index		
1 (immediate)	2628	(1.9)
2 (emergent)	16,908	(12.5)
3 (urgent)	65,917	(48.7)
4 (semi-urgent)	41,007	(30.3)
5 (non-urgent)	9010	(6.7)
Vital signs		
Temperature (F), median (IQR)	98.1	(97.6–98.5)
Pulse rate (bpm), median (IQR)	85	(74–97)
Systolic blood pressure (mmHg), standard deviation (SD)	136	(23.2)
Diastolic blood pressure (mmHg), standard deviation (SD)	79	(14.5)
Respiratory rate (per min), median (IQR)	18	(16–20)
Oxygen saturation (%), median (IQR)	98	(97–99)
Common chief complaints		
Musculoskeletal-related complaints	21,499	(15.9)
Gastrointestinal-related complaints	20,947	(15.5)
General complaints (e.g., fever)	20,581	(15.2)
Injuries	16,731	(12.4)
Respiratory-related complaints	13,539	(10.0)
Neurological-related complaints	9828	(7.3)
Urological-related complaints	6869	(5.1)
Psychiatry-related complaints	4379	(3.2)
Treatment-related complaints (e.g., side effects)	3368	(2.5)
Eye and ear-related complaints	2952	(2.2)
Skin-related complaints	2902	(2.1)
Intoxication	1980	(1.5)
Elixhauser comorbidity measures (≥ 1)	18,249	(13.5)
Clinical outcomes		
Critical care outcome*	2782	(2.1)
Hospitalization outcome†	22,010	(16.2)

Data are presented as number (percentage) of visits unless otherwise indicated

Abbreviations: *ED* emergency department, *IQR* interquartile range, *SD* standard deviation

*Direct admission to intensive care unit (ICU) or in-hospital death

† Admission to an inpatient care site or direct transfer to an acute care hospital

**Table 2:** The 20 most common emergency department diagnoses for critical care and hospitalization outcome

Critical care outcome			Hospitalization outcome		
CCS*	Diagnostic category	*n*	CCS*	Diagnostic category	*n*
122	Pneumonia	161	102	Nonspecific chest pain	1836
102	Nonspecific chest pain	161	251	Abdominal pain	900
109	Acute cerebrovascular disease	138	122	Pneumonia	892
108	Congestive heart failure (non-hypertensive)	133	133	Other lower respiratory diseases	732
133	Other lower respiratory diseases	125	108	Congestive heart failure (non-hypertensive)	626
153	Gastrointestinal hemorrhage	101	127	Chronic obstructive pulmonary disease and bronchiectasis	570
106	Cardiac dysrhythmias	95	245	Syncope	556
131	Respiratory failure, insufficiency, and arrest	90	259	Residual codes (unclassified)	554
2	Septicemia	90	657	Mood disorders	535
259	Residual codes (unclassified)	86	197	Skin and subcutaneous tissue infections	531
55	Fluid and electrolyte disorders	82	106	Cardiac dysrhythmias	530
127	Chronic obstructive pulmonary disease and bronchiectasis	70	109	Acute cerebrovascular disease	483
100	Acute myocardial infarction	64	153	Gastrointestinal hemorrhage	466
101	Coronary atherosclerosis and other heart disease	62	159	Urinary tract infections	463
50	Diabetes mellitus with complications	57	55	Fluid and electrolyte disorders	459
233	Intracranial injury	53	659	Schizophrenia and other psychotic disorders	391
242	Poisoning by other medications and drugs	45	101	Coronary atherosclerosis and other heart disease	319
251	Abdominal pain	43	660	Alcohol-related disorders	285
245	Syncope	40	252	Malaise and fatigue	270
159	Urinary tract infections	40	246	Fever of unknown origin	258

Abbreviation: *CCS* Clinical Classification Software

*The principal diagnoses (> 14,000 *ICD-9-CM* diagnosis codes) were consolidated into 285 mutually exclusive diagnostic categories using the Agency for Healthcare Research and Quality Clinical Classifications Software (CCS) [50], as done previously [51]

### Predicting critical care outcome

**Fig. 1:**
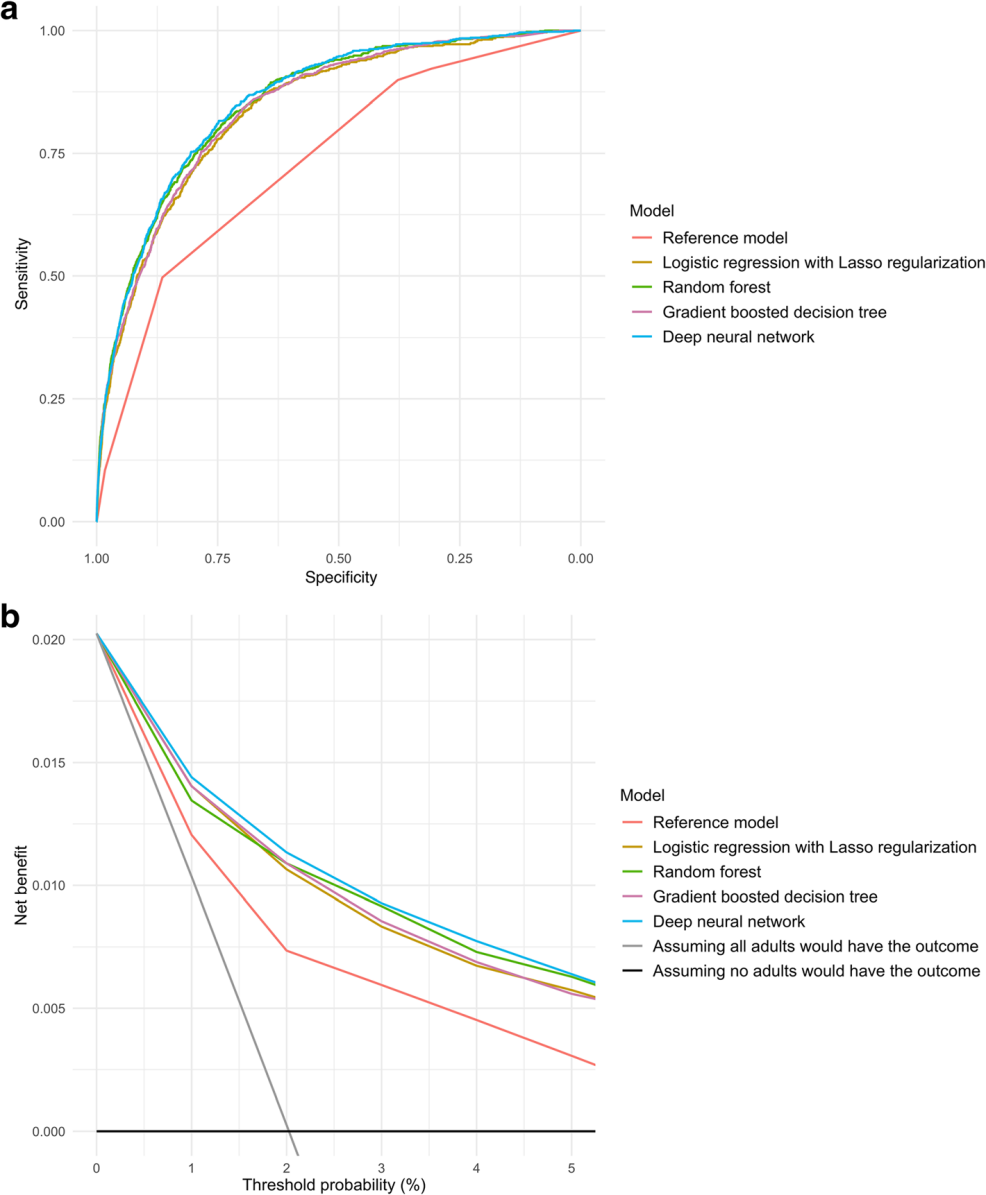
Prediction ability of the reference model and machine learning models for intensive care use and in-hospital mortality in the test set. **a** Receiver-operating-characteristics (ROC) curves. The corresponding values of the area under the receiver-operating-characteristics curve (AUC) for each model are presented in Table 2. **b** Decision curve analysis. *X*-axis indicates the threshold probability for critical care outcome and *Y*-axis indicates the net benefit. Compared to the reference model, the net benefit for all machine learning models was larger over the range of clinical threshold

In the prediction of critical care outcome, the discriminatory abilities of all models are shown in Fig. 1a and Table 3. Compared with the reference model, all four machine learning models demonstrated a significantly higher AUC (all *P* < 0.001). For example, compared to the reference model (AUC 0.74 [95%CI 0.72–0.75]), the AUC was higher in the gradient boosted decision tree (0.85 [95%CI 0.83–0.86]) and deep neural network (0.86 [95%CI 0.85–0.87]) models. Likewise, compared with the reference model, all machine learning models also achieved significant net reclassification improvement (e.g., *P* < 0.001 in the deep neural network model).

**Table 3:** Prediction performance of the reference and machine learning models in the test set

Outcome and model	AUC	*P* value*	NRI†	*P* value†	Sensitivity	Specificity	PPV	NPV
Critical care outcome								
Reference model	0.74 (0.72–0.75)	Reference	Reference	Reference	0.50 (0.47–0.53)	0.86 (0.82–0.87)	0.07 (0.05–0.08)	0.988 (0.988–0.988)
Lasso regression	0.84 (0.83–0.85)	< 0.001	0.39 (0.32–0.46)	< 0.001	0.75 (0.72–0.78)	0.77 (0.75–0.80)	0.06 (0.06–0.07)	0.993 (0.993–0.994)
Random forest	0.85 (0.84–0.87)	< 0.001	0.07 (0.003–0.14)	0.04	0.86 (0.83–0.88)	0.68 (0.68–0.71)	0.05 (0.05–0.06)	0.996 (0.996–0.996)
Gradient boosted decision tree	0.85 (0.83–0.86)	< 0.001	0.32 (0.25–0.38)	< 0.001	0.75 (0.73–0.79)	0.77 (0.75–0.80)	0.06 (0.06–0.07)	0.993 (0.993–0.994)
Deep neural network	0.86 (0.85–0.87)	< 0.001	0.73 (0.67–0.79)	< 0.001	0.80 (0.77–0.83)	0.76 (0.73–0.78)	0.06 (0.06–0.07)	0.995 (0.994–0.995)
Hospitalization outcome								
Reference model	0.69 (0.68–0.69)	Reference	Reference	Reference	0.87 (0.86–0.87)	0.42 (0.39–0.43)	0.23 (0.22–0.23)	0.94 (0.94–0.94)
Lasso regression	0.81 (0.80–0.81)	< 0.001	0.53 (0.50–0.55)	< 0.001	0.71 (0.70–0.72)	0.76 (0.75–0.77)	0.36 (0.35–0.37)	0.93 (0.93–0.93)
Random forest	0.81 (0.81–0.82)	< 0.001	0.66 (0.63–0.68)	< 0.001	0.77 (0.76–0.78)	0.71 (0.70–0.72)	0.34 (0.33–0.35)	0.94 (0.94–0.94)
Gradient boosted decision tree	0.82 (0.82–0.83)	< 0.001	0.63 (0.61–0.66)	< 0.001	0.75 (0.73–0.76)	0.75 (0.74–0.76)	0.37 (0.36–0.38)	0.94 (0.94–0.94)
Deep neural network	0.82 (0.82–0.83)	< 0.001	0.68 (0.65–0.70)	< 0.001	0.79 (0.78–0.80)	0.71 (0.69–0.72)	0.35 (0.34–0.36)	0.95 (0.94–0.95)

Abbreviations: *AUC* area under the curve, *NRI* net reclassification improvement, *PPV* positive predictive value, *NPV* negative predictive value

*P value was calculated to compare the area under the receiver-operating-characteristics curve (AUC) of the reference model with that of each machine learning model

†We used continuous NRI and its *P* value

Additionally, compared with the reference model, all machine learning models demonstrated a higher sensitivity—e.g., 0.50 [95%CI 0.47–0.53] in the reference model vs. 0.86 [95%CI 0.83–0.88] in the random forest model; Table 3. As a trade-off, the specificity of the reference model appeared higher than that of machine learning models—e.g., 0.82 [95%CI 0.82–0.86] in the reference model vs. 0.68 [95%CI 0.68–0.71] in the random forest model. Given the low prevalence of the critical care outcome, all models had high negative predictive values—e.g., 0.988 [95%CI 0.988–0.988] in the reference model vs. 0.996 [95%CI 0.996–0.996] in the random forest model. The AUC of the physiologic score-based model was 0.75 [95%CI 0.74–0.77]. Other predictive performance measures included sensitivity of 0.68 [95%CI 0.65–0.71] and specificity of 0.72 [95%CI 0.71–0.72].

With regard to the number of actual and predicted outcomes stratified by ESI level (Table 4), the reference model correctly predicted critical care outcomes in the triage levels 1 and 2 (immediate and emergent: 49.6% of all critical care outcomes). However, it also over-triaged a large number of patients in these high-acuity categories and failed to predict all critical care outcomes in the levels 3 to 5—i.e., under-triaging 50.4% of critically ill patients. In contrast, the machine learning models successfully predicted 71.3–81.6% of the actual outcomes in the triage levels 3 to 5. Likewise, the decision curve analysis (Fig. 1b) also demonstrated that the net benefit of all machine learning models surpassed that of the reference model throughout the threshold ranges, indicating machine learning-based prediction would more accurately identify patients at high risk with taking the trade-off with over-triages into consideration.

**Table 4:** The number of actual and predicted outcomes of prediction models in the test set

Conventional 5 triage levels (ESI)	Actual number of critical care outcome, *n* (%)	Reference model		Lasso regression		Random forest		Gradient boosted tree		Deep neural network	
		Number of correctly identified outcome	Number of predicted outcome	Number of correctly identified outcome	Number of predicted outcome	Number of correctly identified outcome	Number of predicted outcome	Number of correctly identified outcome	Number of predicted outcome	Number of correctly identified outcome	Number of predicted outcome
1: Immediate (*n* = 768)	86 (11.1)	86	768	72	366	79	460	74	373	76	393
2: Emergent (*n* = 5046)	323 (6.4)	323	5046	241	2175	290	2970	249	2165	264	2387
3: Urgent (*n* = 19,700)	331 (1.7)	0	0	244	5395	269	7482	239	5278	255	5698
4: Semi-urgent (*n* = 12,344)	64 (0.5)	0	0	45	1498	52	2283	44	1464	46	1505
5: Non-urgent (*n* = 2783)	19 (0.7)	0	0	15	308	17	457	14	300	16	315
Overall (*n* = 40,641)	823 (2.0)	409	5814	617	9742	707	13,652	620	9580	657	10,298
Conventional 5 triage levels (ESI)	Actual number of hospitalization outcome, *n* (%)	Reference model		Lasso regression		Random forest		Gradient boosted tree		Deep neural network	
		Number of correctly identified outcome	Number of predicted outcome	Number of correctly identified outcome	Number of predicted outcome	Number of correctly identified outcome	Number of predicted outcome	Number of correctly identified outcome	Number of predicted outcome	Number of correctly identified outcome	Number of predicted outcome
1: Immediate (*n* = 768)	319 (41.5)	319	768	213	393	259	460	241	434	252	471
2: Emergent (*n* = 5046)	1810 (35.9)	1810	5046	1398	2702	1500	3039	1482	2875	1563	3188
3: Urgent (*n* = 19,700)	3628 (18.4)	3628	19,700	2528	7320	2716	8307	2626	7512	2791	8484
4: Semi-urgent (*n* = 12,344)	717 (5.8)	0	0	466	2103	509	2584	488	2154	524	2515
5: Non-urgent (*n* = 2783)	173 (6.2)	0	0	105	434	121	577	117	441	120	547
Overall (*n* = 40,641)	6647 (16.4)	5757	25,514	4710	12,952	5105	14,967	4954	13,416	5250	15,205

Abbreviations: *ESI* Emergency Severity Index, *ICU* intensive care unit

### Predicting hospitalization outcome

**Fig. 2:**
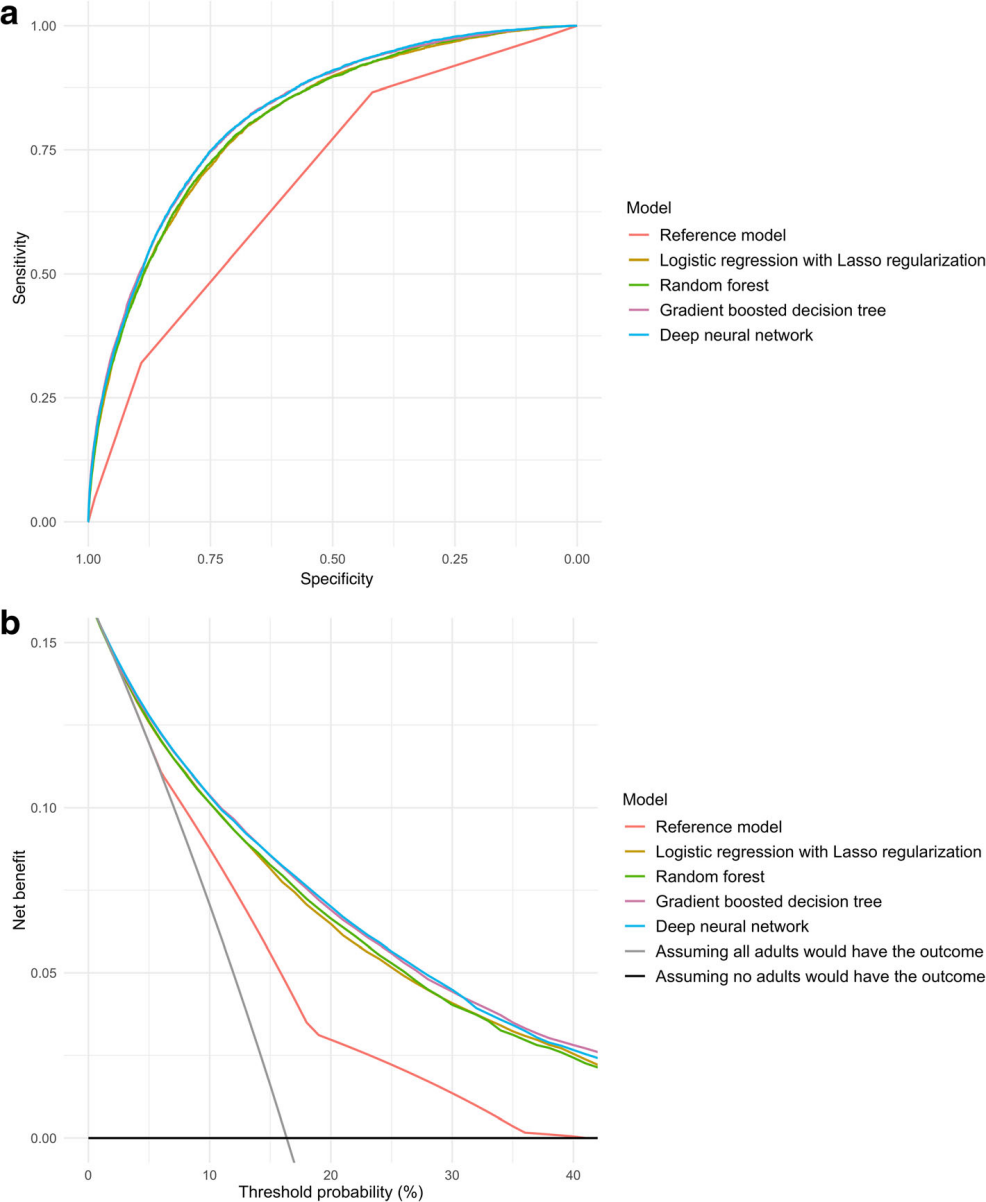
Prediction ability of the reference model and machine learning models for hospitalization in the test set. **a** Receiver-operating-characteristics (ROC) curves. The corresponding values of the area under the receiver-operating-characteristics curve (AUC) for each model are presented in Table 2. **b** Decision curve analysis. *X*-axis indicates the threshold probability for hospitalization outcome and *Y*-axis indicates the net benefit. Compared to the reference model, the net benefit for all machine learning models was larger over the range of clinical threshold

In the prediction of hospitalization outcome, the discriminatory abilities of models are shown in Fig. 2a and Table 3. Compared with the reference model, all four machine learning models demonstrated a significantly higher AUC (*P* < 0.001). For example, compared to the reference model (AUC 0.69 [95%CI 0.68–0.69]; Table 3), the AUC was higher in the gradient boosted decision tree (0.82 [95%CI 0.82–0.83]) and deep neural network (0.82 [95%CI 0.82–0.83]) models. Likewise, compared with the reference model, all machine learning models achieved significant net reclassification improvement (e.g., *P* < 0.001 in the random forest model).

While all the machine learning models demonstrated a lower sensitivity (e.g., 0.87 [95%CI 0.86–0.87] in the reference model vs. 0.71 [95%CI 0.70–0.72] in Lasso regression, Table 3), they yield a higher specificity (e.g., 0.42 [95%CI 0.39–0.43] in the reference model vs. 0.76 [95%CI 0.75–0.77] in Lasso regression model). The AUC of the physiologic score-based model was 0.71 [95%CI 0.71–0.72]. Other predictive performance measures included sensitivity of 0.63 [95%CI 0.62–0.65] and specificity of 0.69 [95%CI 0.68–0.69].

With regard to the number of actual and predicted outcomes stratified by ESI (Table 4), the reference model over-triaged a large number of patients in the triage levels 1 to 3 and failed to predict all hospitalization outcomes in the levels 4 and 5—i.e., under-triaging 13.4% of hospitalized patients. In contrast, the machine learning models successfully predicted 64.2–72.4% of the actual outcomes in the levels 4 and 5. Likewise, the decision curve analysis (Fig. 2b) also demonstrated that the net benefit of all machine learning models surpassed that of the reference model throughout the threshold ranges.

### Variable importance

**Fig. 3:**
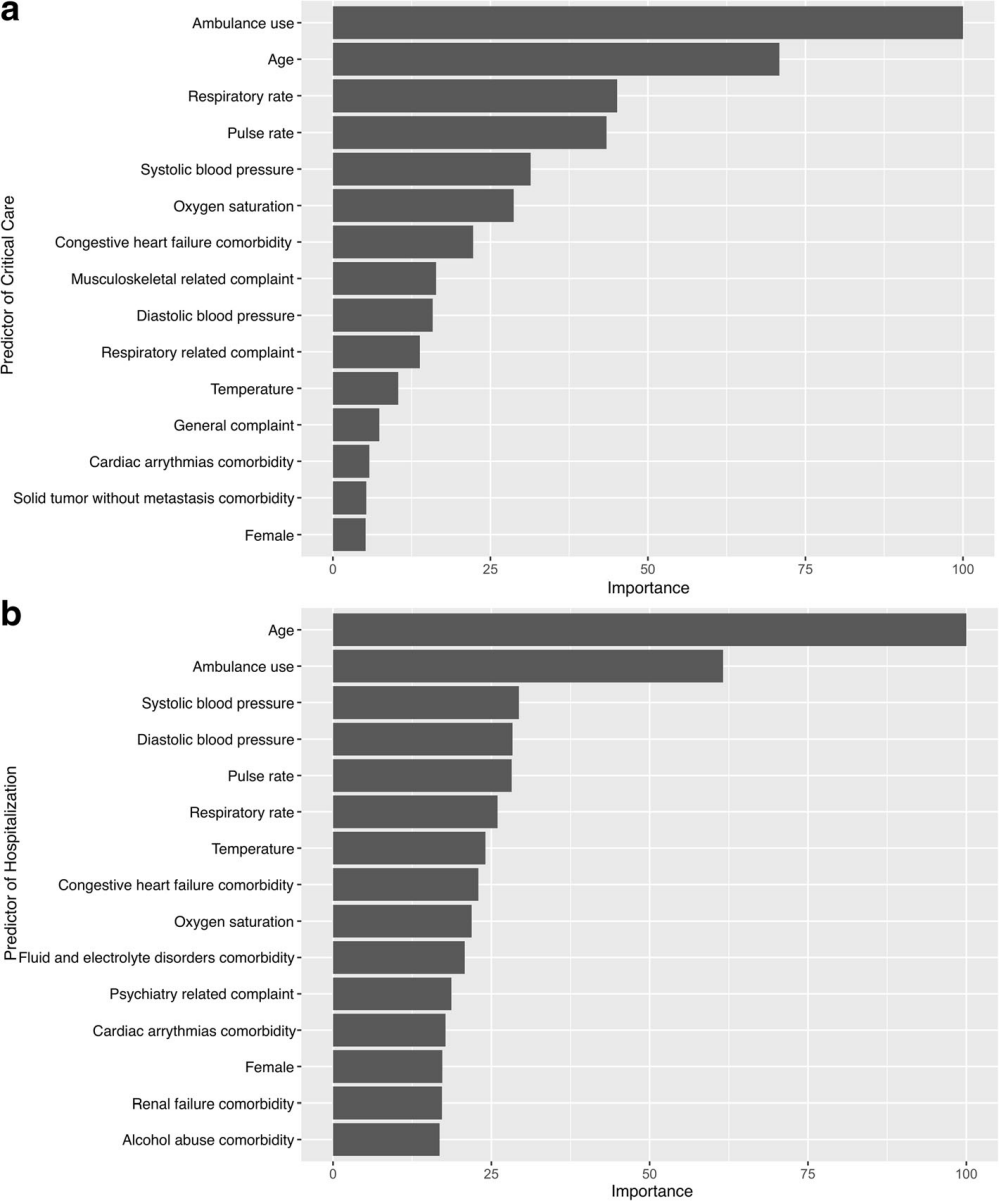
Variable importance of predictors in the random forest models. The variable importance is a scaled measure to have a maximum value of 100. The predictors with a variable importance of the top 15 are shown. **a** Critical care outcome. **b** Hospitalization outcome

**Fig. 4:**
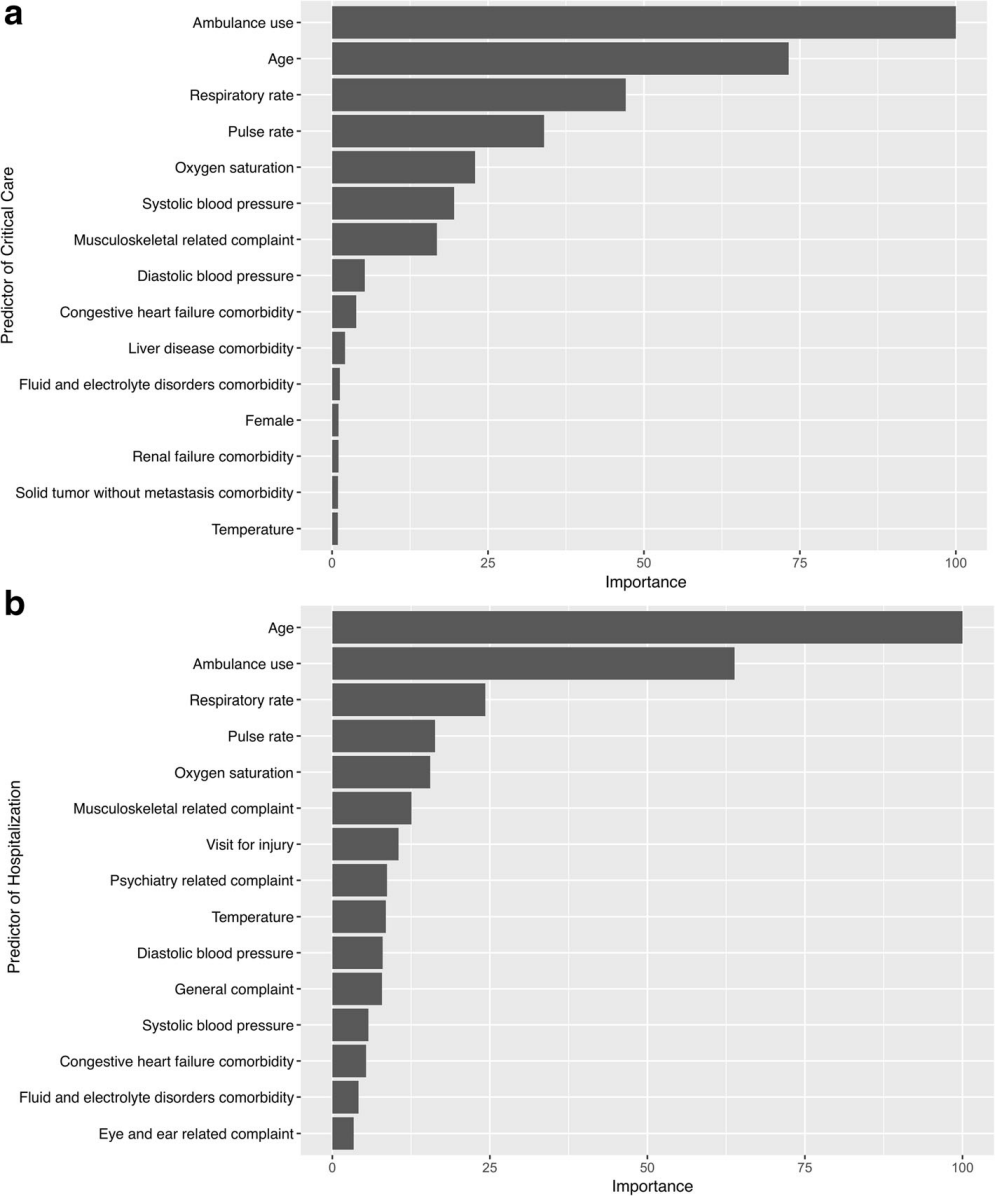
Variable importance of predictors in the gradient boosted decision tree models. The variable importance is a scaled measure to have a maximum value of 100. The predictors with a variable importance of top 15 are shown. **a** Critical care outcome. **b** Hospitalization outcome

To gain insights into the relevance of each predictor, Figs. 3 and 4 summarize the 15 most important predictors of random forest and gradient boosted decision tree models for each outcome. In the random forest models, ambulance use, age, vital signs, and comorbidities (e.g., congestive heart failure) were most important predictors for the critical care (Fig. 3a) and hospitalization (Fig. 3b) outcomes. The variable importance was similar in the gradient boosted decision tree models **(**Fig. 4).

## Discussion

Based on the data of 135,470 adult ED visits, we applied four modern machine learning approaches (i.e., Lasso regression, random forest, gradient boosted decision tree, and deep neural network) to the routinely available triage information. Compared to the conventional model, based on ESI algorithm [8], these machine learning models demonstrated a superior performance in predicting critical care and hospitalization outcomes, including improved AUCs and net reclassification. Additionally, the machine learning models had a higher sensitivity for the critical care outcome with a reduced number of under-triaged critically ill patients, and had a higher specificity for the hospitalization outcome with fewer over-triages. Moreover, the decision curve analysis revealed that all machine learning models yielded a larger net benefit—the trade-off between appropriate triages and over-triages—throughout the wide range of thresholds. To date, this is the first investigation that has comprehensively examined the utility of modern machine learning models for predicting clinical outcomes in a large population of adult patients in the ED.

The major goals of ED triage are to accurately differentiate high-risk patients from more-stable patients and to efficiently allocate finite ED resources. Prior studies have documented that current triage algorithms (e.g., ESI) have a suboptimal predictive ability to identify critically ill patients, low inter-rater agreement, and high variability within a same triage level [9–13]. While the use of a complete set of information—such as detailed data on past and present illnesses, physical examinations, and repeated measurements during the ED course—may improve prediction abilities, it is impractical at ED triage settings because of the limited information and time available. An alternative approach to enhance clinicians’ prediction abilities is to utilize advanced machine learning models. Recently, the machine learning models have been applied to outcome predictions in diverse medical fields—e.g., mortality in patients with sepsis [14], cardiac complications in patients with acute chest pain [43], rehospitalization in patients with congestive heart failure [44], critical care and hospitalization outcomes in children [18] and in adults with asthma and COPD exacerbation [19], and unplanned transfer to ICU [15]. The current study corroborates the promise suggested by these recent studies and extends them by demonstrating superior predictive abilities of modern machine learning models over the conventional model in a large population of adults in the ED.

ED triage systems seek for an optimal balance between under-triages and over-triages. The present study showed that, compared to the conventional ESI approach, our machine learning models demonstrated a higher sensitivity in predicting ICU admission and in-hospital mortality. Indeed, the models correctly identified critically ill patients who would be inappropriately under-triaged into lower-acuity ESI levels (levels 3 to 5), supporting the advantages of machine learning-based prediction at the ED triage where rapid identification of patients at high risk is paramount. By contrast, patients who are going to be admitted do not always need excessive recourse in the ED (e.g., patients with cellulitis who are admitted to an ED observation unit). Therefore, predictions of hospitalization outcome using a high-sensitivity (and low-specificity) model would lead to over-triages and excessive resource utilization. However, our machine learning models yielded a higher specificity in predicting hospitalization with a reduced number of over-triaged patients, particularly in the higher-acuity ESI levels (levels 1 to 3), who may not utilize excessive resource. Additionally, the utility of machine learning-based prediction is further buttressed by the greater net benefit observed in the decision curve analysis—which incorporates the trade-off between over- and under-triages [39, 45]—across the wide range of clinical thresholds.

The reasons for the improvement in predictive abilities observed in the machine learning models are likely multifactorial. First, the ESI algorithm heavily relies on subjective clinical assessment of anticipated ED resource use that leads to modest performance and large variabilities between providers [8–10]. Second, advanced machine leaning approaches are adept at handling high-order interactions between the predictors and non-linear relationships with the outcome [17, 28]. Third, while overfitting in conventional models is often problematic, our machine learning models adopted multiple rigorous approaches to mitigate overfitting, such as regularization, cross-validation, and dropout. Although our machine learning models achieved the superior predictive ability, the performance was not perfect. This is attributable, at least partly, to the limited set of predictors, subjectivity of data (e.g., visit reasons), various clinical factors after ED triage (e.g., quality and timeliness of ED management and patients’ clinical responses), differences in patients’ health behaviors, providers’ practice patterns, and availability of ED resources. Yet, in the era of health information technology, machine learning-based prediction has a scalable advantage—e.g., updating prediction models through an automated extraction of electronic health record data and integration with digital images, natural language processing, and continuous monitoring of physiological data [46–48]. This scalability had been unattainable in the conventional models where decisions were made based on fixed rules encoding knowledge. Taken together, our findings and recent developments suggest that machine learning approaches are indispensable next-generation assistive technology to further advance clinical decision-making abilities [49].

The current study has several potential limitations. First, we excluded samples with missing information. Yet, the analytic and non-analytic cohorts were generally comparable in the patient demographics, ED presentation, and outcomes. These similarities argue against substantial selection bias. Second, the quality of data is important in data-driven machine learning-based prediction. Although survey data may have some misclassification and ascertainment bias, NHAMCS has a coding error rate of < 1% in their 10% quality control sample [22]. Third, NHAMCS data do not collect some helpful clinical variables (e.g., chronic medications, socioeconomic status, health behaviors). However, the goal of the present investigation is not to develop prediction models using a broad set of predictors but to derive machine learning models using a limited set of predictors that are *routinely* available at current ED triage settings. Finally, the indication and clinical threshold of ICU admission, hospitalization, and hospital transfer depend on the local healthcare resource and may vary between different EDs and clinicians. However, the decision curve analysis demonstrated that the net benefit of all machine learning models was consistently greater than that of the reference model across the wide range of threshold probabilities (or clinical preferences). This finding supports the generalizability of prediction models.

## Conclusions

Based on the analysis of 135,470 adult ED visit data, we developed the machine learning models using ED triage data. These models yielded a superior performance in predicting critical care and hospitalization outcomes over the conventional ESI-based model. Particularly, the machine learning models would reduce the number of critically ill who are under-triaged by the conventional approach. Furthermore, the models would also decrease over-triaging hospitalization outcomes that lead to excessive resource allocation to less-sick patients. Moreover, the machine learning models also yielded a greater net benefit across wide ranges of threshold probabilities. While external validations are necessary, the current study lends substantial support to the application of machine learning-based predication to ED triage as a decision support technology. Machine learning models—as assistive technologies—offer new avenues for enhancing the clinician’s ED triage decision making, which will, in turn, improve patient care and optimize resource utilization in already-stressed emergency care systems.

## Additional file

Additional file 1: Comparison of predictor variables and outcomes between the analytic and non-analytic cohort. (DOCX 16 kb)

## Abbreviations

AUC: Area under the receiver-operating-characteristics curve
CDC: Centers for Disease Control and Prevention
ED: Emergency department
ESI: Emergency Severity Index
NHAMCS: National Hospital and Ambulatory Medical Care Survey
NPV: Negative predictive value
PPV: Positive predictive value
TRIPOD: Transparent Reporting of a multivariable prediction model for Individual Prognosis Or Diagnosis
