# Supplementary material for: Emergency department triage prediction of clinical outcomes using machine learning models
Source: Crit Care. 2019 Feb 22;23:64. doi: 10.1186/s13054-019-2351-7 (PMC6387562; doi:10.1186/s13054-019-2351-7)
Supplement: Supplementary file 1 — Comparison of predictor variables and outcomes between the analytic and non-analytic cohort. (DOCX 16 kb) [file 13054_2019_2351_MOESM1_ESM.docx]

**Additional file 1. Comparison of predictor variables and outcomes between the analytic and non-analytic cohort**

| **Variables** | | **Analytic cohort** | | **Non-analytic cohort** | | |
| --- | --- | --- | --- | --- | --- | --- |
|  | | n=135,470 | | n=67,883 | | missing  (%) |
| Age (year), median (IQR) | | 46 | (29-60) | 43 | (29-59) | 0 |
| Female sex | | 58,450 | (43.1) | 29,678 | (43.7) | 0 |
| Mode of arrival | |  |  |  |  |  |
|  | Ambulance | 26,820 | (19.8) | 11,326 | (19.5) | 9,876 (14.5) |
| Vital signs | |  |  |  |  |  |
|  | Temperature (F), median (IQR) | 98.1 | (97.6-98.5) | 98.1 | (97.6-98.6) | 9,922 (14.6) |
|  | Pulse rate (bpm), median (IQR) | 85 | (74-97) | 86 | (74-96) | 12,936 (19.1) |
|  | Systolic blood pressure (mmHg), standard deviation (SD) | 136 | (23.2) | 133 | (23.3) | 6,521 (9.6) |
|  | Diastolic blood pressure (mmHg), standard deviation  (SD) | 79 | (14.5) | 78 | (14.5) | 6,793 (10.0) |
|  | Respiratory rate (per min), median (IQR) | 18 | (16-20) | 18 | (16-20) | 9,874 (14.5) |
|  | Oxygen saturation (%), median (IQR) | 98 | (97-99) | 98 | (96-99) | 25,776 (38.0) |
| Common chief complaints | |  |  |  |  |  |
|  | Musculoskeletal-related complaints | 21,499 | (15.9) | 10,599 | (15.6) | 0 |
|  | Gastrointestinal-related complaints | 20,947 | (15.5) | 9,588 | (14.1) | 0 |
|  | General complaints (e.g., fever) | 20,581 | (15.2) | 8,954 | (13.2) | 0 |
|  | Injuries | 16,731 | (12.4) | 9,434 | (13.9) | 0 |
|  | Respiratory-related complaints | 13,539 | (10.0) | 5,778 | (8.5) | 0 |
|  | Neurological-related complaints | 9,828 | (7.3) | 4,448 | (6.6) | 0 |
|  | Urological-related complaints | 6,869 | (5.1) | 3,729 | (5.5) | 0 |
|  | Psychiatry-related complaints | 4,379 | (3.2) | 2,940 | (4.3) | 0 |
|  | Treatment-related complaints (e.g., side effects) | 3,368 | (2.5) | 2,269 | (3.3) | 0 |
|  | Eye and ear-related complaints | 2,952 | (2.2) | 1,521 | (2.2) | 0 |
|  | Skin-related complaints | 2,902 | (2.1) | 1,464 | (2.2) | 0 |
|  | Intoxication | 1,980 | (1.5) | 894 | (1.3) | 0 |
| Elixhauser comorbidity measures (≧1) | | 18,249 | (13.5) | 8,405 | (12.4) | 0 |
| Emergency Severity Index | |  |  |  |  | 32,147 (47.4) |
| 1 (immediate) | | 2,628 | (1.9) | 1,486 | (4.2) |  |
| 2 (emergent) | | 16,908 | (12.5) | 4,655 | (13.0) |  |
| 3 (urgent) | | 65,917 | (48.7) | 15,932 | (44.6) |  |
| 4 (semi-urgent) | | 41,007 | (30.3) | 10,100 | (28.3) |  |
| 5 (non-urgent) | | 9,010 | (6.7) | 3,563 | (10.0) |  |
| Clinical outcomes | |  |  |  |  |  |
|  | Critical care outcome* | 2,782 | (2.1) | 1,332 | (2.0) | 0 |
|  | Hospitalization outcome† | 22,010 | (16.2) | 10,143 | (14.9) | 0 |

Abbreviations: ED, emergency department; IQR, interquartile range; SD, standard deviation

* Direct admission to intensive care unit (ICU) or in-hospital death

† Admission to an inpatient care site or direct transfer to an acute care hospital
